# Using Duchscher's Theory of Transition Shock To Inform The Experience Of Newly Graduated Nurses In Qatar: A Qualitative Case Study

**DOI:** 10.15694/mep.2021.000156.1

**Published:** 2021-06-04

**Authors:** Tricia Tieleman, Stuart Cable

**Affiliations:** 1 Sidra Medical and Research Center; 2 University of Dundee

**Keywords:** Transition Experience, Qatari Nursing, New Graduate Nurse, Qualitative Case Study

## Abstract

This article was migrated. The article was marked as recommended.

Background

The transition experience of new graduate nurses (NGN) is a complex and stressful period of adjustment and adaptation. The transition period is often negative leading to job dissatisfaction and increased intent to leave. Literature concerned with transition experiences of NGNs in the Middle East is lacking, where the educational, healthcare and cultural context are dissimilar to those in the published literature.

Aim

To explore the transition experiences of Qatari NGNs to inform development of a formal transition to practice program at a specialty hospital.

Methods

A qualitative case study design was conducted to explore the professional role transition experiences of Qatari NGNs. Data was collected through the Casey-Fink Graduate Experience Survey, and an unstructured focus group.

Results

Qatari NGNs experienced challenges of professional role adaptation and feeling ill-prepared in terms of competency and job-readiness, resulting in self-doubt, frustration and fear. Desires to fit-in were negatively impacted by language, discrimination and lack of social support, and professional identity development.

Conclusions

Professional role transition of Qatari NGNs involves a complex interplay of intrapersonal, interpersonal, organizational and cultural factors. Experiences can be enhanced by accessible, nurturing, multi-level social support to facilitate socialization and improve clinical practice competency. Managing expectations by preparing students for transition and providing transitional support during their first year of practice will improve their experience. Duchscher’s Theory of Transition Shock offers insights into the transition expereinces that can inform this support.

## Introduction

### Introduction

The complexities and challenges of professional role transition experienced by new graduate nurses (NGNs) have been well documented in North American nursing literature for four decades. Limited data exists describing NGN experience in other countries. According to Western literature, the first two years of practice is a time of adaptation, integrating theory with practice, and developing clinical competence, with many studies discussing experiences, challenges, stresses and support needed during this transition ( Dwyer and Hunter Revell, 2016). Significant to this study is the applicability of past findings to a Middle Eastern cultural context of nursing education and practice ( Al-Dossary, Kitsantas and Maddox, 2016).

### Background/Literature review

Nursing practice in Qatar is predominantly led by expatriate nurses. Qatari NGNs are inducted into a clinically experienced expatriate nursing workforce and the orientation is targeted at experienced nurses, unsuitable to the needs of NGNs, which could negatively impact on quality of patient care ( Fielden, 2012). Furthermore, the nursing profession in Gulf countries is not highly regarded in society ( Maben *et al.,* 2010) creating discord between idealistic constructs of the nursing profession, the reality of the healthcare culture and wider social context.

Duchscher (2009) identified that NGNs entering professional practice encounter a wide range of changes, illustrated in her theory of Transition Shock. This describes a process of adjustment, motivated and mediated by changing roles, responsibilities, relationships, and levels of knowledge in personal and professional lives. Central to this experience is differences between known educational context, requirements of professional practice and the interplay of emotional, physical, socio-cultural/developmental and intellectual influences leading to feelings of loss, doubt, disorientation and confusion ( Duchscher, 2009).

Dwyer and Hunter Revell (2016) identified a complex interplay between intrapersonal, interpersonal and organizational influences in NGN transition. Educational preparation and psychological capital significantly influence experiences, promote positive self concept and establish social skills which facilitate the transition process. Transition is positively influenced by personal attributes of resilience, emotional intelligence, flexibility and adaptability ( Parker *et al.,* 2014).

Alboliteeh, Magarey, and Wiechula (2018) reported language barriers as a stressor for Saudi NGNs with English as a second language. Poor language skills affected professional communication and provision of nursing care and negatively impacted on opportunities for learning and advancement. Ability to speak and comprehend language of others facilitates understanding of culture and verbal communication is critical in discerning intentional meaning of cultural concepts ( O’Hagan *et al.,* 2014).

Support from colleagues, peers or senior nurses directly impacts job satisfaction, intention to leave and turnover ( Dwyer and Hunter Revell, 2016). Trained preceptors are critical to successful transition of NGNs playing a role in building confidence, promoting connections to proficient nurses as role models and realizing the professional role within the context of the interprofessional team ( Laschinger *et al.,* 2016). A study of NGNs by Clark and Springer (2012) found that welcoming staff make NGNs feel like they matter, fit in and integrate with the team, consistent with the findings in Middle Eastern literature. In the Kingdom of Saudi Arabia (KSA), NGNs described lack of acceptance, not being welcomed into the workplace, being ignored, and feelings of isolation ( Alboliteeh, Magarey and Wiechula, 2017). Omani NGNs reported expatriate nurses formed cliques resulting in poor collegial relationships, suggesting that ‘Omanisation’ generated a perceived threat to job security for expatriates creating tension and repudiation ( Al Awaisi, Cooke and Pryjmachuk, 2015). Saudi NGNs felt poorly understood by expatriate managers and reported their cultural background and needs were not considered ( Alboliteeh, Magarey, and Wiechula, 2018).

Socialization is described extensively as influential on successful transition. Accounts of NGNs witnessing or experiencing nurse to nurse intimidation and aggressive language are common ( Thomas and Magilvy, 2011) and incivility is linked with decreased empowerment, job satisfaction, increased burnout and attrition ( Laschinger *et al.,* 2016). Hostile workplaces inhibit NGNs from asking questions and pursuing learning opportunities, potentially decreasing self-confidence and motivation ( Thomas and Magilvy, 2011). Being part of a good team where NGNs experience civility and consistent, active support helps diminish feelings of fear and stress ( Halpin, Terry and Curzio, 2017). Alboliteeh, Magarey, and Wiechula, (2018) reported Saudi NGNs experienced harsh treatment from expatriate nurses and other colleagues, resulting in perceptions of unfair treatment and self-doubt.

Organizational influences including safe, supportive work environments, workload, and role expectations impact successful NGN transition. NGNs who perceived their work environment to be healthy or supportive experienced better outcomes, such as decreased emotional exhaustion, and environmental reality shock, and identify experienced nurses and nurse leaders as a fundamental support ( Dwyer and Hunter Revell, 2016). Negative workplace experiences depleted coping resources, resulting in work-life imbalance and interference ( Laschinger *et al.,* 2016). Workload and inadequate staffing stressors contribute to adverse outcomes including perceptions of job demands and difficulties, job dissatisfaction and emotional exhaustion ( Dwyer and Hunter Revell, 2016). The emotive consequences of burnout at work carries over into personal lives of NGNs leading to increased attrition ( Laschinger *et al.,* 2016).

The KSA and Omani studies found poor image of the nursing profession, and social customs and values significantly influenced the NGN experience. Nursing in both countries is perceived as “dirty, low status work because it entails handling others’ bodies and dealing with bodily fluids” ( Al Awaisi, Cooke and Pryjmachuk, 2015), creating discord between an image of nursing learned in school and perceptions of society. In Gulf countries nursing has low status devaluing the education and training offered and the care nurses provide, which is further compounded by traditional views and a belief that it is “wrong for girls to work in nursing” because it is “not right for a girl to stay the whole night outside the home” ( Alboliteeh, Magarey, and Wiechula, 2018, pp. 80).

The literature suggests many themes influencing transition experiences of NGNs worldwide, however the cultural context surrounding nursing education and practice in Qatar is a significant additional factor.

### Aim of the study

The aim of this study is to explore the experiences of Qatari NGNs to inform a transitional program at a speciality hospital in Qatar. The primary research question for this study is “What is the transition experience of Qatari NGNs?”

## Methodology

A descriptive case study design was adopted to explore transition experiences of NGNs appropriate to a phenomenon of interest which is complex and highly conceptualized, with multiple variables unsuitable for control ( Yin, 2014). The study sought to uncover accurate perspectives of the personal, organisational and cultural experiences of Qatari NGNs.

### Data Collection: Instruments and Methods

This case study used the Casey-Fink Graduate Nurse Experience Survey designed to explore experiences of North American NGNs ( Casey *et al.,* 2004) in combination with an unstructured focus group to gain deeper insight into the complexities of Qatari NGN transition experiences. The focus group was audiotaped as photography and video recording are not widely accepted among Qatari women.

### Participants and Sampling

Participants were graduates from a baccalaureate nursing program in Qatar with 6 to 24 months of clinical practiceworking clinically in one hospital. Population size was 16, and sample size was n=14. Nursing as a career choice in Qatar is at an early stage, hence numbers of graduates are small, creating limitations in sampling. Convenience sampling was adopted using opportunely accessible NGNs as participants ( Polit and Beck, 2006). Participants were sent an e-mail link to an anonymous survey. A focus group was scheduled within one month from initiation of recruitment to minimize attrition. Participants were welcomed and time was alloted for greetings, showing respect for cultural norms, thereby faciliating a prompt start to the session. To respect the participant’s religious needs, the focus group was deliberately held between two obligatory prayer times.

### Data Analysis

Survey data analysis consisted of scoring items for frequency and emerging themes of responses did not reveal significant trends within this study, but was used to complement inferences drawn from the qualitative analysis of the focus group ( Vaismoradi, Turunen and Bondas, 2013).

Focus group data was analyzed thematically for commonalities, relationships, patterns, theoretical constructs or explanatory principles ( Vaismoradi, Turunen and Bondas, 2013). Recorded data was transcribed verbatim, checked against audio for accuracy and participants were provided transcripts to verify accuracy ( Dicicco-Bloom and Crabtree, 2006). Initial codes were generated using a transcript copy giving each item equal consideration. Initial codes were collated into potential themes. Themes were reviewed, checked against the original data set and examined for internal coherence, consistency and distinctiveness ( Braun and Clarke, 2006).

### Ethical Considerations

Ethical approval was obtained by the hospital’s Institutional Review Board (IRB) prior to the onset of the study. Adherence to IRB protocols included maintaining accurate records, ensuring that ethical principles were upheld throughout the study. Consent to participate was obtained using an approved consent form that provided information about the study, any possible risks, how data would be used and outlined the “right to withdraw” at any time in both English and Arabic.

## Findings and Discussion

Following systematic data analysis findings were collated under three main themes that reflected data constructs described in Duchscher’s (2009) Theory of Transition Shock:

• Navigating role transition
• Navigating workplace culture
• Navigating the cultural landscape

### Navigating Role Transition

As NGNs transition from academia to professional practice, they experience a theory-practice gap and challenges with achieving work-life balance. Qatari NGNs expressed feeling inadequately prepared for their clinical role, difficulties applying theoretical knowledge to practice and concerns that their inexperience may cause them to harm patients. Duchscher indicates amalgamating theoretical knowledge with what is seen and done in the ‘real world’ as a primary task of transitioning NGNs. Qatari NGNs reported, “ *They always give you stable patients, we’ve only seen the normal, so we cannot recognize the abnormal” (NG5).* *“If you don’t have the answer maybe you can look it up, but you cannot understand how to relate it to the patient” (NG5).* NGNs frequently express concerns about their ability to notice that which was outside the norm and limited tacit and practical knowledge contribute to feelings of self-doubt ( Duchscher, 2009). Data in this study illustrated similar concerns, *“I was thinking...what am I going to do?” (NGN5).* Their critical thinking and reasoning lacked the depth and breadth that comes with experience, requiring purposeful and gradual progression of clinical accountability and independent practice ( Duchscher, 2009).

Realization the safety net of the academic setting is gone, and professional role expectations contributed to feeling afraid, *“It’s fear because you’re being held reliable [sic] now. You’re not a student anymore, you’re a nurse.” (NGN8) and “I was scared all the time” (NGN3).* Primary fears experienced by NGNs are “being exposed as clinically incompetent, failing to provide safe care, inadvertently harming their patient, and inability to cope with designated roles and responsibilities” ( Duchscher, 2009, p.1107). Self-trust was reported as a countermeasure among those with more experience as they separate from the dependent role, *“You just have to take a breath and you have to trust yourself.” (NGN6).* This suggests a transformation of self, a socio-developmental task required of the NGN ( Duchscher, 2009).

Imperative to achievement of skill competence is ample opportunity to practice required skills ( Duchscher, 2009) and insufficiency lead to self doubt, reflected in this study. NGNs in this study reported insufficient opportunity to practice skills and procedures. “ *You’re supposed to graduate, enter the institution and get the experience, but people always forget that we need those opportunities to learn and get experience” (NGN8).*

NGNs entered the workforce with expectations of gaining on-the-job experience, clashing with experienced nurses’ expectations of job-readiness, *“I don’t feel safe to say I don’t know, because I know I will be judged (NGN7).* This fear of judgment created pressure to prove themselves. *“I have to fight. I have seen so many experienced nurses in my unit I have now to fight to prove myself” (NG5).* Pfaff *et al.,* (2014) indicated that learning to manage self-imposed expectations of practice and the expectations of other healthcare professionals is a struggle for many NGNs.

Competing demands of professional and personal roles is challenging however for Qatari NGNs, shiftwork incurs additional negative cultural pressures, *“Working 12-hour shifts, a night shift, try explaining that to your grandparents. That’s being Arab and a Muslim and being in a job that goes against your culture, not religion, but culture” (NGN3).*

The issue of working nights and Friday was a prevailing concern due to cultural pressures. Working on Fridays evoked frustration, “ *It’s a challenge for me when I have shifts in the weekends because Friday is always a family gathering.”* *(NGN1).* Friday is the most significant day of the week for Muslims as the day of congregational prayers, one of the most strongly emphasized duties in Islam. Fears of disappointing family, shift rotations and changing social habits and routines creates a sense of loss. Familial obligations and expectations significantly influence achieving work-life balance, *“you have to make time for them. I have to visit my grandparents at least once a week and I have to have lunch with my mom or else it becomes a family issue” (NGN3).* Balancing personal life with professional work is another primary task of transitioning NGNs ( Duchscher, 2009).

Many NGNs experience exhaustion around month 4, largely related to the efforts to stabilize their emotions, an action recognised in Duchscher’s theoretical model. *“I’ve called in for a sick day. I was burned out. I was just exhausted. I just needed a day off.” (NGN3).* Pursuit of acceptance and validation from colleagues inhibits them from requesting time off, *“I feel like I have to prove myself and that asking for vacation makes me seem like I’m weak” (NGN3).* NGNs fear disappointing their colleagues and will go to extremes to mask their feelings of inadequacy to prevent being rejected by their colleagues as valued and contributing members of the professional community ( Duchscher, 2009).

### Navigating Workplace Culture

NGNs struggle to discover professional role identity, master necessary skills, and find their place in organizational culture and assimilate with the professional practice team. Ongoing social support from key people in the workplace is a known influential interpersonal factor in successful NGN transition ( Dwyer and Hunter Revell, 2016). Most NGNs identified being well supported by their peers, *“I had a lot of support and everybody knew I was a fresh grad and I needed that help” (NGN3)* and *“I think I’m really lucky, all of the nurses are very open to me and they are always willing to help me” (NGN6),* though some identified inadequate social support, *“sometimes I go to the station and nobody is there.” (NGN6).* Lack of immediate access to social supports for knowledge, emotional support, practice advice and feedback potentiates feelings of isolation and self-doubt. Lack of such supports negatively influences job satisfaction and intentions to leave ( Dwyer and Hunter Revell, 2016). NGNs described inconsistency with preceptor support and its impact on transition. *“Our preceptors keep changing, this is my second preceptor, now I’m just as lost as I was before” (NGN8).* Duchscher (2009) states insufficient preceptor guidance and support and inadequate feedback on performance contributes to self-doubt and confusion for the NGN.

A designated educator who is accessible, responsive to and supportive of the unique needs of transitioning NGNs mitigates organizational deficiencies ( Parker *et al.,* 2014). Some NGNs spoke about the powerful impact of having an educator dedicated to their transition:

*“You being in our life, you’re like a star in the dark nights. You light this experience by giving us advice and support us and you never give up from us.” (NGN5),* and,

“Having someone whether it’s on the unit or out of the unit care for you and know they’re there for you. That gives a big impact on our experience transitioning as a fresh graduate.” (NGN8)

NGNs desire to be regarded as a professional is influenced by belonging (being part of the team), knowing (ability to answer patient and family questions) and affirmation (feeling valued for what they do) ( Jewell, 2013). Some NGNs described positive integration, *“we are like a family” (NGN3)* but others identified challenges, *“there was no introduction, and nobody even knows I’m a fresh graduate” (NGN3).* Being accepted by the larger professional nursing community is identified as a primary developmental task of the NGN, many yearn to enter the ‘clique’ ( Duchscher, 2009).

Some NGNs described not feeling like a valued member of the team and the resulting negative impact. Clark and Springer, (2012) identified that when NGNs feel they added no value or burdened their preceptors they become dissatisfied with their job and show decreased commitment to the organization.

“I feel very isolated. I don’t feel valued, so no matter what I say it doesn’t matter because it is coming from me. I feel that the staff don’t accept me the way I am” (NGN7)

“It makes you feel that you are worth nothing and nobody cares about you” (NGN5)

This challenge was exacerbated by language, seen as a barrier for integrating with the multinational team, *“it was hard, sometimes I didn’t know what was going on simply because there’s a lot of people who speak one language and they’re speaking that language you don’t understand” (NGN3)* and it makes them feel *“isolated” (NGN7) and “withdrawn” (NGN5).*

Inconsistent information sharing was identified as a barrier, *“they wouldn’t share anything, I would just accidentally know about something” (NGN4).* Challenging intra/interpersonal relations create feelings of doubt and confusion and for NGNs according to Duchscher (2009), lack of control felt by the NGNs intensifies the challenges experienced by multiple languages being spoken, creates a barrier to asking for information, and organizational and unit-based information sharing is not consistent, leaving them feeling lost, isolated and withdrawn.

Qatari NGNs reported negative assumptions of expatriate staff impede their integration. *“There’s this misconception that when you’re born here, you’re rich and you’re spoiled, so when you go into a unit, they already have this misconception about you, and that’s not true.” (NGN3)* *“they think we don’t work so we have to prove them the opposite way” (NGN4).* The consequence of such negativity Duchscher (2009) proposes is hypersensitivity and self-critical states.

Many NGNs reported senior nurse and physician conduct bolstered hierarchical rather than collegial relationships which impacted on the clinical climate. They attributed this challenge to working with staff coming from other institutions, *“most of our residents come from (another hospital) so what they’re used to is completely different from what we’re trying to achieve here” (NGN3).* Navigating the hierarchy did not appear to be a challenge, as all the NGNs reported feeling confident communicating with physicians and nurse leaders. *“I had a conflict, so I had to speak to my manager and actually speak up to her” (NGN8).* This confidence in interprofessional communication they propose was facilitated by supportive relationships with preceptors and organizational leaders through role-modeling collaborative behaviours which supported NGN socialization into the team.

Negative work experiences deplete coping resources resulting in work-life imbalance, a known predictor of intent to leave ( Laschinger *et al.,* 2016) as one powerful testimony illustrated, *“I wanted to leave nursing. Really, I love my job, I love to be a nurse. I had a dream that I was building 5 years ago to reach the point that I am here now, but after facing these issues, I feel that I don’t want to continue, I don’t want to be that nurse I dreamed to be because I don’t have the strength to fight” (NGN5).*

This captures the critical interplay between the components of Duchscher’s (2009) transition conceptual model, perpetuating feelings of loss, disorientation, confusion and doubting nursing as a career choice.

### Navigating the Cultural Landscape

Significant to transition experiences in Qatar is overcoming challenges associated with societal perceptions of the profession, which contributes to role uncertainty and doubts regarding their chosen profession, described in combative terms, *“When you’re a nurse in the Middle East, that’s what you signed up for, you’re fighting the misconceptions of nursing.” (NGN3).* Nursing at its core is a profession based in service, however in Qatar, this negatively influences society’s image of the profession. One NGN shared,

“If you are Arabic, you take a deep breath when you go into a patient’s room, especially if they are from here or the Arab world, and you prepare yourself for the questioning that you will get. ‘Why you choose to become a nurse? You are working 12-hour shifts? What is your salary? From which family are you? Just yesterday someone asked me about my salary” (NGN4)

Another NGN reported being asked *“why did you study to be a maid? You could be working at the bank and dress nice” (NGN6).* Nursing is believed to be a low-paying job illustrated by stories of patients attempting to give nurses money, *“after the patient delivered the baby, the mother she came to me and she put her hand in her bag and she tried to give me 100 riyals” (NG7).* Alboliteeh, Magarey, and Wiechula, (2018) described the cultural perceptions of nursing in KSA as performing menial tasks and providing basic care, tasks viewed as having low status. Many Saudis perceived nursing as a job for maids or uneducated people. Omani NGNS described basic nursing care increased perceptions of nurses as servants, which made them feel embarrassed and ashamed ( Al Awaisi, Cooke, and Pryjmachuk, 2015). Qatari NGNs identified feeling culpable for not working hard enough to change the image believing that performing basic tasks reinforces the perceptions *,*

“The problem that makes it more difficult and harder, they ask you to clean the floor, they ask you to make the coffee and so this is what the patient sees. They don’t know the other things that nurses do, and to blame nurses and ourselves also, you know, for getting the coffee” (NG7)

They described attempting to positively change the image of nursing though professional practice and educating patients and families, *“you try to speak to them, to educate them that we are doing things different. I think patients and families see that now” (NGN3).* These NGNs were passionate about being change agents, describing ideals and aspirations for their careers inconsistent with the current view,

“We have to fight double as hard as people from western places because we are the ones actually setting what nursing is here. Our generation of graduates, we’re the ones actually changing things” (NGN3)

They spoke ardently about required support from expatriate nurses and nursing leadership to change the image of nursing in Qatar.

“It’s hard to change the image, to show our patients, to show the population what nursing really is” (NG7).

*“We want to change, and we need someone to support us, if somebody is not helping us, we will not make it” (NGN1).*

Despite challenges, they expressed satisfaction with providing nursing care in their language, as Arabic speaking nurses are a minority,

“There aren’t many Arabic speaking nurses, so it creates a therapeutic communication between you and the patient. They feel like they can talk to you, and at the end of it, which for me makes nursing so worth it, every time I’m with a patient at the end, and they say, ‘my experience was so different with you’. It makes me feel warm inside and cuddly.” (NGN3)

According to Duchscher’s (2009) conceptual model, positive experiences and relationship building with patients and families provide the NGNs with reassurance, validation and positive reinforcement which can mitigate feelings of loss and confusion related to their professional role and identity.

## Conclusions

This study reflects how Qatari NGNs experienced the contradictions between roles, responsibilities, relationships, knowledge, and performance expectations. Their experiences resonate with findings in the Western and Middle Eastern literature. Qatari NGNs reported difficulties applying theoretical knowledge to clinical experiences resulting in feeling unprepared for a professional role, and apprehensiveness in responding to and managing deteriorating patient status and emergency response procedures.

Role transition of Qatari NGNs is a complicated, stressful period critically affected by the complex interplay of intrapersonal, interpersonal, organizational and cultural factors. The NGNs experienced fear, self doubt and immense pressure to prove themselves as competent, exacerbated by limited opportunities to practice skills and procedures and expectations of colleagues to be job ready. Lack of consistent support, difficulties integrating with colleagues, adjusting to shiftwork, balancing personal obligations and negative cultural view of nursing creates dissatisfaction with their chosen profession and most notably, intent to leave nursing altogether. They feel empowered to influence the cultural view of nursing in Qatar, however the need for support from the expatriate nurses was critical to their success.

Duchscher (2009) recommends incorporating professional role transition topics like stages of transition and the experience of transition shock, intergenerational nursing, inter/intra-professional communication, workload delegation and management, lifestyle adjustment, conflict management and professional roles and responsibilities. Strong evidence supports the benefit in incorporating professional role transition topics to assist NGN preparation. Improved preparation of nursing students for the professional role could be achieved through increased collaboration between hospital and academic institutions and provision of formal pre-graduate transition workshops, internships and student practicum placement opportunities. Transition experiences can be enhanced by accessible, nurturing, multi-level social support to facilitate socialization and improve clinical practice competency. Managing expectations by preparing students for role transition and providing transitional support by a dedicated educator during the first year of practice positively influence the transition.

## Take Home Messages

• Role transition of Qatari new graduate nurses in complicated and is critically affected by intrapersonal, interpersonal, organizational and cultural factors.
• Transition experiences of new graduate nurses in Qatar are strongly affected by societal perceptions of the nursing profession.
• New graduate nurses in Qatar experience challenges integrating with a largely expatriate nursing workforce.
• Professional role adaptation for new graduate nurses in Qatar are impacted by language barriers, discrimination and lack of social supports

## Notes On Contributors

### Tricia Tieleman, RN, MMED, BScN

A passionate nurse educator with more than 10 years of experience in both academic and professional nursing education in Canada and the Middle East. She been a part of the project teams for two new specialty children’s hospital projects in the Middle East, establishing pediatric simulation programs, developing inter-professional orientation programs, nursing competency assessment and professional practice programs such as preceptor development and a new graduate nurse residency program.

At the time of this study, Tricia was employed full-time as the Nurse Educator for the New Graduate Nurse Program at Sidra Medical Research Center in Doha, Qatar and completing her Masters in Medical Education via distance learning at the University of Dundee, Dundee, Scotland. This study was her student research project.

### Dr. Stuart Cable

Current role is the design, development and delivery of Master’s in Medical Education. Supporting postgraduate students with dissertations and theses. Range of scholarly activities - policy, practice and research. Particular interest in contemplative practices to support higher education and compassionate practice. At the time of this study, Dr. Cable was Tricia’s faculty supervisor at the University of Dundee, Dundee, Scotland. Dr. Cable supervised this student research project.
