# Supplementary material for: Using Duchscher's Theory of Transition Shock To Inform The Experience Of Newly Graduated Nurses In Qatar: A Qualitative Case Study
Source: MedEdPublish (2016). 2021 Jun 4;10:156. [Version 1] doi: 10.15694/mep.2021.000156.1 (PMC10939564; doi:10.15694/mep.2021.000156.1)
Supplement: Peer review history

# Reviewer report: Reviewer response for version 1

**DOI:** 10.21956/mep.18921.r27048

**Published:** 2021-10-10

**Authors:** Ken Masters

**Affiliations:** 1 Sultan Qaboos University

This review has been migrated. The reviewer awarded 5 stars out of 5 A really interesting qualitative case study using Duchscher's theory of Transition Shock to understand the experience of newly graduated nurses in Qatar.The paper begins with a solid background review of the relevant literature, both international and regional. The results from the focus group have been well-situated in Duchscher's theoretical framework.Overall, the paper provides valuable insights into the issues facing the newly-graduated nurses, and highlights the complexities of the professional and cultural environments.Some small points of criticism:• From the description, it appears that only one focus group was held. With n=14, this does reduce the amount of participation in the group. For future studies, I would strongly recommend a reduction in the focus group size, to a maximum of 10. (12 can be done, but that is pushing limits, and 14 is problematic). So, in this case, two groups of 7 each would have been better.• The Methodology should state the length (time) of the focus group (excluding greetings, etc).• The Methodology should also indicate which authors did the theming, verification, and whether or not any software was used.Nevertheless, the paper is a fine read, and a valuable contribution to the field. Well done.

Reviewer Expertise:

NA

No decision status is available

# Reviewer report: Reviewer response for version 1

**DOI:** 10.21956/mep.18921.r27049

**Published:** 2021-07-14

**Authors:** Richard Hays

**Affiliations:** 1 James Cook University

This review has been migrated. The reviewer awarded 3 stars out of 5 This is a nicely done small qualitative study of the transition to the workplace of local nurses trained in one Gulf region country. While student-workplace transitions always pose challenges, they may be more noticeable in health care, particularly for doctors and nurses. The findings show that there are additional challenges in this particular country due to cultural rather than religious beliefs. I note that both authors are expats and so I wonder how much this reflects 'outsider' thinking? If a revision is intended I would suggest three additional issues be included to add strength. The first is the international literature on the transitional anxiety that is common at the student-workplace interface be included in the background and literature review. The second is that some analysis or reaction from local healthcare managers may strengthen the findings and improve local engagement in moving forward. The third is that some ideas for how to improve the transition, possibly including facilitation of cultural change, should be mentioned. Addressing these comments may help guide change and may merit an additional star.

Reviewer Expertise:

NA

No decision status is available
